# *Helicobacter pylori* in the Indonesian Malay’s descendants might be imported from other ethnicities

**DOI:** 10.1186/s13099-021-00432-6

**Published:** 2021-06-04

**Authors:** Ari Fahrial Syam, Langgeng Agung Waskito, Yudith Annisa Ayu Rezkitha, Rentha Monica Simamora, Fauzi Yusuf, Kanserina Esthera Danchi, Ahmad Fuad Bakry, Arnelis, Erwin Mulya, Gontar Alamsyah Siregar, Titong Sugihartono, Hasan Maulahela, Dalla Doohan, Muhammad Miftahussurur, Yoshio Yamaoka

**Affiliations:** 1 Division of Gastroenterology, Department of Internal Medicine, Faculty of Medicine-Cipto Mangunkusumo Teaching Hospital, University of Indonesia, Jakarta, Indonesia; 2 Faculty of Medicine, Universitas Airlangga, Surabaya, Indonesia; 3 Institute of Tropical Disease, Universitas Airlangga, Surabaya, Indonesia; 4 Department of Internal Medicine, Faculty of Medicine, University of Muhammadiyah Surabaya, Surabaya, Indonesia; 5 Department of Internal Medicine, Faculty of Medicine, Universitas Airlangga, Surabaya, Indonesia; 6 Division of Gastroentero-Hepatology, Department of Internal Medicine, Faculty of Medicine, Dr. Zainoel Abidin General Hospital, Universitas Syiah Kuala, Banda Aceh, Indonesia; 7 Department of Internal Medicine, Dr. M Thomsen Nias Gunungsitoli General Hospital, Nias, Indonesia; 8 Division of Gastroentero-Hepatology, Department of Internal Medicine, Faculty of Medicine, Sriwijaya University, Palembang, Indonesia; 9 Division of Gastroenterology, Department of Internal Medicine, Faculty of Medicine, Andalas University, Padang, Indonesia; 10 Department of Internal Medicine, Cimacan General Hospital, Cianjur, Indonesia; 11 Division of Gastroentero-Hepatology, Department of Internal Medicine, Faculty of Medicine, University of Sumatera Utara, Medan, Indonesia; 12 Division of Gastroentero-Hepatology, Department of Internal Medicine, Faculty of Medicine-Dr Soetomo Teaching Hospital, Universitas Airlangga, Jalan Mayjend Prof. Dr. Moestopo No. 6-8, Surabaya, 60286 Indonesia; 13 Department of Environmental and Preventive Medicine, Oita University Faculty of Medicine, 1-1 Idaigaoka, Hasama Machi, Yufu City, Oita, 879-5593 Japan; 14 Department of Medicine, Gastroenterology and Hepatology Section, Baylor College of Medicine, Houston, TX USA

**Keywords:** *Helicobacter pylori*, Malays, Prevalence, Population genetics, Sumatra, Epidemiology

## Abstract

**Background:**

Even though the incidence of *H. pylori* infection among Malays in the Malay Peninsula is low, we observed a high *H. pylori* prevalence in Sumatra, which is the main residence of Indonesian Malays. *H. pylori* prevalence among Indonesian Malay descendants was investigated.

**Results:**

Using a combination of five tests, 232 recruited participants were tested for *H- pylori* and participants were considered positive if at least one test positive. The results showed that the overall *H. pylori* prevalence was 17.2%. Participants were then categorized into Malay (Aceh, Malay, and Minang), Java (Javanese and Sundanese), Nias, and Bataknese groups. The prevalence of *H. pylori* was very low among the Malay group (2.8%) and no *H. pylori* was observed among the Aceh. Similarly, no *H. pylori* was observed among the Java group. However, the prevalence of *H. pylori* was high among the Bataknese (52.2%) and moderate among the Nias (6.1%). Multilocus sequence typing showed that *H. pylori* in Indonesian Malays classified as hpEastAsia with a subpopulation of hspMaori, suggesting that the isolated *H. pylori* were not a specific Malays *H. pylori*.

**Conclusions:**

Even though the ethnic groups live together as a community, we observed an extremely low *H. pylori* infection rate among Indonesian Malay descendants with no specific Indonesian Malay *H. pylori*. The results suggest that *H. pylori* was not originally among these groups and *H. pylori* was imported from other ethnic groups.

## Background

*Helicobacter pylori* infects approximately half of the human population, but its prevalence varies among countries. Variations in *H. pylori* prevalence are influenced by several factors, including the virulence of *H. pylori*, geographical location, the culture of the host, and host ethnicity [1]. A meta-analysis study of *H. pylori* infection showed that the highest prevalence was found in Africa (79.1%), South America (63.4%), and Asia (54.7%) [2]. Interestingly, *H. pylori* infection prevalence is low in certain ethnic groups, despite having the same environmental exposure as other ethnic groups [3].

Malays, members of the Austronesian family, are an ethnic group who speak the Malayo-Polynesian language [4, 5]. Malays predominantly inhabit the South-East Asia region, especially the Malay Peninsula, east coast of Sumatra, and the coast of Borneo. According to the “Taiwan” theory, the Malays originated from Taiwan and migrated to the Malay Peninsula through the Philippines and Borneo approximately 1,500 years ago, although they might have simultaneously traveled alongside people originating from Yunnan, China [6, 7]. After reaching the Malay Peninsula, the Malays began to spread to Indonesia (predominantly Sumatra), several areas of Borneo, and the western tip of Java. Although Sumatra is predominantly occupied by Indonesian Malays, several ethnicities are classified as Proto-Melayu, including the Bataknese and Nias ethnic groups, which are considered older ancestors of the modern Indonesian Malays [8].

Other ethnic groups also reside in Indonesia, including the Javanese and Sundanese. The Javanese reside mainly on Java Island. Javanese and Sundanese have very similar cultures, languages, and cuisines. Importantly, the Sundanese reside almost exclusively in the western part of Java. The origin, history, and language of Peninsular Malays, Javanese, and Sundanese are very similar. In addition to the Melayu-Minang and Melayu-Bugis, who are sub-ethnic Malays in Malaysia, there is a sub-ethnic group named Melayu-Jawa, who have a close genetic relationship to the Indonesian population, including the Javanese [9]. These findings indicate that Javanese and Sundanese might have a common ancestral and cultural history with Peninsular Malays [4]. Therefore, Javanese and Sundanese are considered Indonesian Malay ethnic descendants.

To date, native inhabitants of several areas of Indonesia are still categorized as part of the Malay ethnic group, although they are divided into many sub-ethnic groups. This was reinforced by a population census conducted by the Dutch colonial government in 1930. The 1930 population census (volkstelling) used anthropological studies, language approaches, geography, history, and ethnography to determine ethnic groups; thus, many researchers use the 1930 census data by the Dutch government as a reference for tribal composition in Indonesia [10]. In the 1930 census, populations inhabiting Sumatra island, parts of Borneo, and the western tip of Java were still categorized as ethnic Malay. These ethnicities are still used with some extensions.

The *H. pylori* infection rate among Malays in Malaysia was only 19.6% and was significantly lower than the Chinese and Indian populations [11]. In contrast, the Javanese had a very low *H. pylori* infection rate of only 2.4% [12]. In addition, our previous data on the five largest islands in Indonesia showed a high *H. pylori* prevalence in Sumatra, the main residence of Indonesian Malays. Since there is a close relationship between Peninsular Malays and Indonesian Malays, we hypothesized that the *H. pylori* infection rate among Indonesian Malay descendants would be lower than the prevalence in Malaysia. Herein, we examined *H. pylori* prevalence among Indonesian Malay ethnic descendants.

## Results

### Sample demographic characteristics

A total of 232 samples from 126 males and 106 females were analyzed. Participants were recruited from Banda Aceh (n = 38), Medan (n = 22), Dolok Sanggul (n = 47), Padang (n = 33), and Palembang (n = 38) on Sumatra island, Gunungsitoli (n = 32) on Nias Island, and Cimacan (n = 22) on Java Island. The mean age of the participants was 45.54 ± 14.64 and ranged from 17–83 years old. According to the ethnicity, 37 (15.9%) participants were Aceh, 67 (28.8%) participants were Bataknese, 4 (1.7%) participants were Javanese, 36 (15.5%) participants were Malay, 33 (14.2%) participants were Minang, 33 (14.2%) participants were Nias, and 22 (9.5%) participants were Sundanese. The ethnicity and history suggest that the Bataknese and Nias are older ethnicties than the current Malay ethnic group and are considered part of the Proto-Malay people [8]. Javanese are considered descendants of the Malays as part of the Austronesian expansion. Thus, we separated the dataset into an ethnic Malay group (Aceh, Malay, and Minang), an ethnic Java group (Javanese and Sundanese), ethnic Bataknese, and ethnic Nias. After separation into four groups, no significant differences in age and sex were detected between the groups (*P* = 0.322 and *P* = 0.321, respectively).

### Clinical outcome observations

Normal mucosa was observed in 136/232 (58.6%) participants, 61/232 (26.3%) had gastritis, 34/232 (14.7%) had peptic ulcer disease, and 1/232 (0.4%) had gastric cancer. Older participants had significantly more severe gastric mucosal conditions (*r* = 0.207, *P* = 0.001). No significant differences in gastric mucosal conditions were detected between male and female participants (*P* = 0.373). Gastric conditions were associated with ethnic groups (*P* = 0.002). *H. pylori* infection was significantly associated with worse gastric conditions compared with non-infected individuals (*P* < 0.001) (Table 1).

**Table 1:** Clinical outcome observations

Variables	Normal (%)	Gastritis (%)	PUD (%)	Cancer (%)
Mean Age (SD)	43.0 (14.0)	49.1 (15.8)	49.4 (13.3)	45^a^
Sex				
Male	79 (58.1)	29 (47.5)	18 (52.9)	0 (0.0)
Female	57 (41.9)	32 (52.5)	16 (47.1)	1 (100)
*H. pylori* infection^b^	7 (5.15)	23 (37.7)	10 (29.4)	0 (0.0)

PUD, peptic ulcer disease; ^a^, number of patients was only one, the SD value could not be calculated; ^b^, this number was calculated based on at least one test positive consideration

### Low *H. pylori *infection in the ethnic Malay group

Overall *H. pylori* infection was extremely low in the ethnic Malay group. *H. pylori* measured using a rapid urease test (RUT) was detected in 2/106 (1.9%) participants, by immunohistochemistry (IHC) in 1/106 (0.94%), by histology in 0/106 (0.00%), by enzyme-linked immunosorbent assay (ELISA) in 1/106 (0.94%), and by culture in 1/106 (0.94%) (Table 2). The overall *H. pylori* prevalence based on at least one positive test was 3/106 (2.83%) participants. The infected participants tended to be older than the uninfected participants (56 vs 42, *P* = 0.095). The three infected individuals were from Padang (1 subject) and Palembang (2 subjects) and no significant differences in prevalence between locations were detected (*P* = 0.337). *H. pylori* positivity was not associated with sex (*P* = 0.814).

**Table 2:** *H. pylori* infection among Malay ethnic groups

Characteristics	Mean Age (SD) (yrs)	Sex (%)		*H. pylori* Positivity (%)					
		Male	Female	At least one	RUT	Histology	IHC	ELISA	Culture
Overall	42.52 (14.5)	60 (56.59)	46 (43.41)	3 (2.83)	2 (1.89)	0 (0.00)	1 (0.94)	1 (0.94)	1 (0.94)
Malay Group Ethnics									
Aceh	37.5 (13.4)	20 (54.1)	17 (45.9)	0 (0.00)	0 (0.00)	0 (0.00)	0 (0.00)	0 (0.00)	0 (0.00)
Malay	44.5 (13.0)	21 (58.3)	15 (41.7)	2 (5.56)	1 (2.78)	0 (0.00)	1 (2.78)	1 (2.78)	1 (2.78)
Minang	45.9 (16.3)	19 (57.6)	14 (42.4)	1 (3.03)	1 (3.03)	0 (0.00)	0 (0.00)	0 (0.00)	0 (0.00)
Locations									
Banda Aceh	37.4 (13.2)	20 (52.6)	18 (47.4)	0 (0.00)	0 (0.00)	0 (0.00)	0 (0.00)	0 (0.00)	0 (0.00)
Padang	46.3 (16.1)	20 (60.6)	13 (39.4)	1 (3.03)	1 (3.03)	0 (0.00)	0 (0.00)	0 (0.00)	0 (0.00)
Palembang	44.4 (13.2)	20 (57.1)	15 (42.9)	2 (5.71)	1 (2.86)	0 (0.00)	1 (2.86)	1 (2.86)	1 (2.86)

*RUT* rapid urease test, *IHC* immunohistochemistry, *ELISA* enzyme-linked immunosorbent assay

### No H. pylori observed in the Java ethnic group

No *H. pylori* was detected in the Java ethnic group, similar to our observation among the Malay ethnic group, using any of the five different tests. The occurrence of *H. pylori* was 0/26 (0.00%) using RUT, IHC, histology, ELISA, or culture tests (Table 3).

**Table 3:** *H. pylori* in Java ethnic groups

Characteristics	Mean Age (SD)	Sex (%)		*H. pylori* Positivity (%)					
		Male	Female	At least one	RUT	Histology	IHC	ELISA	Culture
Overall	43.6 (12.9)	20 (76.88)	6 (23.12)	0 (0.00)	0 (0.00)	0 (0.00)	0 (0.00)	0 (0.00)	0 (0.00)
Java Group Ethnic									
Javanese	41.8 (8.9)	2 (50.00)	2 (50.00)	0 (0.00)	0 (0.00)	0 (0.00)	0 (0.00)	0 (0.00)	0 (0.00)
Sundanese	44.0 (13.7)	18 (81.78)	4 (18.22)	0 (0.00)	0 (0.00)	0 (0.00)	0 (0.00)	0 (0.00)	0 (0.00)

*RUT* rapid urease test, *IHC* immunohistochemistry, *ELISA* enzyme-linked immunosorbent assay

### Origin of H. pylori in Indonesian Malays

**Fig. 1:**
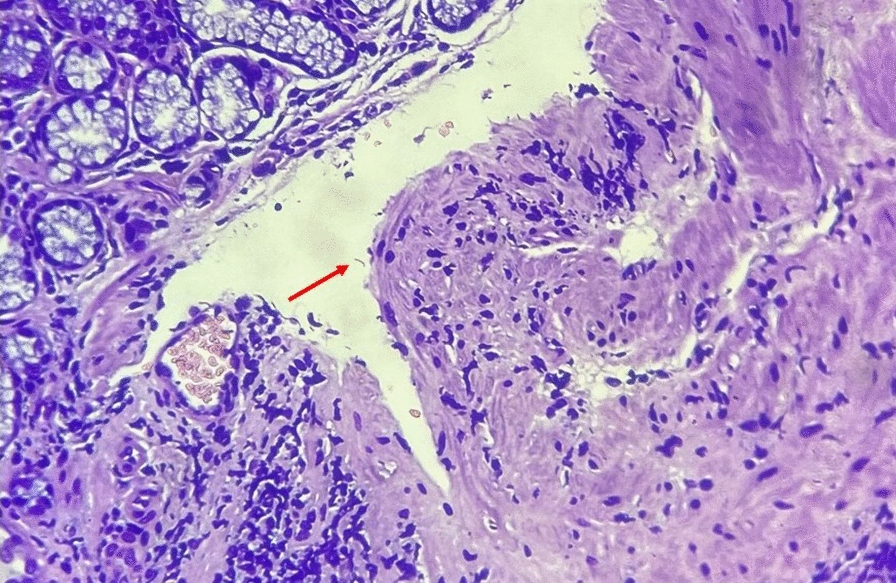
Histological examination of a *H. pylori* positive case. The representative image of a *H. pylori* positive case in our current study. The red arrow indicates the observed *H. pylori*

Among all Indonesian Malay participants, we could only detect *H. pylori* in one participant and we could only isolate one sample. We performed a population genetic analysis of *H. pylori* from only one sample isolated from an Indonesian Malay originating from Padang city. Using no-admixture model of STRUCTURE analysis, the *H. pylori* sample belonged to the hpEastAsia population (Fig. 1A). Subsequent analysis comparing only the hpEastAsia population showed that the sample belonged to the hspMaori subpopulation (Fig. 1B). A phylogenetic tree analysis showed that this strain is located near Indonesian Minahasanese [13] and Melanesians hspMaori [14].

### Incidence of H. pylori high among Bataknese and moderate among ethnic Nias

Separately, we analyzed *H. pylori* positivity among Bataknese. *H. pylori* prevalence among the ethnic Bataknese was considerably higher compared with the prevalence in the Malay or Java ethnic groups, based on every diagnostic method. The highest positivity rate was measured using the RUT (37.8%), followed by ELISA (26.9%), IHC (25.4%), culture (20.8%), and histology (19.4%) (Table 4). Based on having at least one *H. pylori* positive test, 35/67 (52.2%) participants tested positive for *H. pylori*; the prevalence in the Bataknese was significantly higher compared with the prevalence in the Malay ethnic group (*P* < 0.001), despite the two groups living in similar locations on Sumatra island. As expected, the Malay ethnic group had significantly lower odds for *H. pylori* infection compared to the Bataknese ethnic group by 0.049-fold (95% confidence intervals [CI] = 0.014–0.167, *P* < 0.001). *H. pylori*-infected individuals were significantly older than uninfected individuals (53.7 vs 46.6, *P* = 0.019). No significant association between sex and *H. pylori* prevalence was detected. Location analysis showed Medan tended to have a higher *H. pylori* infection rate than Dolok Sanggul (75.0% vs 40.3%, *P* = 0.08).

**Table 4:** *H. pylori* prevalence among Bataknese and Nias ethnic groups

Characteristic	Mean Age (SD)	Sex (%)		*H. pylori* Positivity (%)					
		Male	Female	At least one	RUT	Histology	IHC	ELISA	Culture
Ethnicities									
Bataknese	51.7 (14.9)	32 (47.78)	35 (52.23)	35 (52.23)	25 (37.28)	13 (19.39)	17 (25.41)	18 (26.89)	14 (20.88)
Nias	44.2 (12.2)	14 (42.38)	19 (57.62)	2 (6.06)	2 (6.06)	1 (3.03)	1 (3.03)	1 (3.03)	1 (3.03)
Locations									
Medan	51.1 (13.8)	10 (50.0)	10 (50.0)	15 (75.0)	14 (70.0)	0 (0.0)	1 (5.0)	0 (0.0)	4 (20.0)
Dolok Sanggul	51.2 (15.6)	21 (44.7)	26 (55.3)	20 (40.3)	11 (23.4)	13 (27.7)	16 (34.0)	18 (38.3)	10 (21.3)
Gunungsitoli	44.5 (12.2)	14 (43.8)	18 (56.2)	2 (6.3)	2 (6.3)	1 (3.1)	1 (3.1)	1 (3.1)	1 (3.1)

*RUT* rapid urease test, *IHC* immunohistochemistry, *ELISA* enzyme-linked immunosorbent assay

We observed *H. pylori* in only 2 (6.1%) participants in the Nias ethnic group (positive in at least test). Two (6.1%) Nias participants tested positive using RUT, 1 (3.03%) using ELISA, 1 (3.03%) using IHC, 1 (3.03%) using culture, and 1 (3.03%) using histology. The prevalence of *H. pylori* in the Nias ethnic group was significantly lower than the prevalence in the Bataknese (6.1% vs 52.2%, *P* < 0.001) but not significantly different from the ethnic Malay group.

### Performance of each diagnostic test when H. pylori prevalence varied

**Fig. 2:**
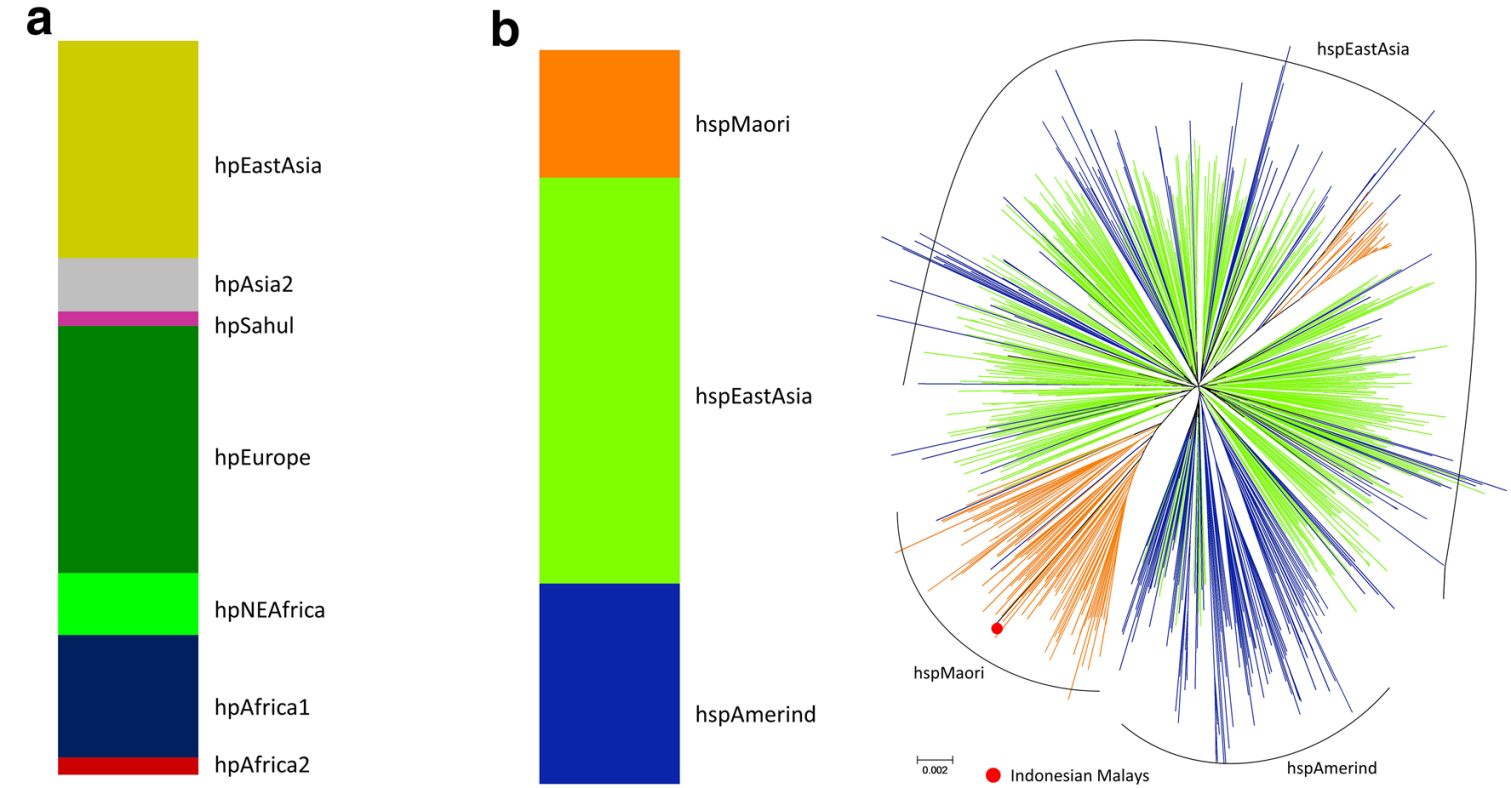
Population genetics of Indonesian Malay *H. pylori*. **A** Among globally available *H. pylori* MLST data in pubMLST database, the analysis yielded seven populations, denoted as hpAfrica2, hpAfrica1, hpNEAfrica, hpEurope, hpAsia2, hpSahul, and hpEastAsia. The isolated Indonesian Malay *H. pylori* was classified as hpEastAsia. **B** In a subsequent analysis using only hpEastAsia as the database, Indonesian Malay *H. pylori* was categorized as hspMaori subpopulation on both the phylogenetic and STRUCTURE analyses

Overall, we observed a good essential agreement (91.9%) between tests with a fair κ-coefficient value of 0.719 (*P* < 0.001). After we divided the participants into different ethnic groups, we found a high essential agreement of 97.5% and a fair κ-coefficient value (0.293, *P* < 0.001) among the Malay ethnic group. In addition, we found a good essential agreement of 72.3% and substantial κ-coefficient value 0.669 (*P* < 0.001) among Bataknese (Fig. 2).

Even though we found good inter-diagnostic method consistency, there was a major difference in invasiveness between the tests. We classified the diagnostic tests as invasive (RUT and culture) or non-invasive (ELISA). Then, we examined the performance of invasive versus non-invasive in at least one positive scenario for each group compared to histology/IHC as the positive group. The invasive test yielded a fair performance with sensitivity and specificity values of 78.9% and 92.5%, respectively, and overall accuracy of 91.4% (Table 5). The non-invasive test performed well, with an overall accuracy of 96.2%, a sensitivity of 83.3%, and a specificity of 97.4%. These results suggest that the non-invasive test was better for diagnostic testing in this population.

**Table 5:** Test performance based on invasive and non-invasive classification

Parameters	Invasive	Non-invasive
Overall accuracy (95% CI)	91.4% (87.0–94.7)	96.2% (92.6–98.3)
Sensitivity (95% CI)	78.9% (54.4–93.9)	83.3% (58.6–96.4)
Specificity (95% CI)	92.5% (88.1–95.7)	97.4% (94.3–99.1)
NPV (95% CI)	98.1% (95.3–99.2)	98.4% (95.7–99.4)
PPV (95% CI)	48.4% (35.7–61.3)	75.0% (55.2–87.9)

## Discussion

This study described the *H. pylori* prevalence among the Malay descendant ethnic groups who reside in Sumatra and Java. We observed an extremely low *H. pylori* prevalence among the Malay ethnic group, consisting of Aceh, Malay, and Minang. Furthermore, we did not find any *H. pylori* among the Aceh. Our observations were similar to a multi-racial study of *H. pylori* infection in Malaysia and Singapore that found a low *H. pylori* infection rate among Malay people [15–18]. This low prevalence among Malay ethnic groups is probably due to several factors, including host polymorphisms, agent virulence, and environmental factors (such as culture, food, and habit). A single-nucleotide polymorphism analysis revealed the presence of several gene polymorphisms among Malays compared to Chinese and Indian, which may contribute to the low *H. pylori* infection rate [19, 20]. As for the pathogen virulence, the virulence may be low in indigenous Malay *H. pylori,* resulting in easier detachment from the gastric mucosa [21]. There are several *H. pylori* virulence factors related to colonization ability, including blood group antigen-binding adhesin, SabA, outer inflammatory protein, and *H. pylori* outer membrane protein Q [22]. The activity of these proteins may be associated with lower colonization in Indonesian Malay *H. pylori*. In addition, Malays add and consume “pegaga” (*Centella asiatica*) in their daily food and this consumption is significantly associated with lower *H. pylori* prevalence [23]. *Centella asiatica* may increase gastric mucus production [24], leading to lower *H. pylori* colonization. Consumption of “dadih”, a traditional fermented buffalo milk, is common among Indonesian Malays. In addition, Indonesian Malays commonly consume *Zanthoxylum acanthopodium*, spicy flavored fruits [25]. Both *Zanthoxylum acanthopodium* and “dadih” have anti-microbial activity against several gram-positive and gram-negative pathogenic bacteria [26, 27]. Therefore, consumption of these foods may prevent *H. pylori* infection.

We also found an extremely low prevalence of *H. pylori* among the Java ethnic group; in particular, no *H. pylori* was detected in the Sundanese. Sundanese is a specific ethnic group residing mostly in the western part of Java. The Sudanese share a similar language, culture, and food with the Javanese, the most predominant ethnicity in Indonesia. This finding supports our previous results showing that the Javanese have a low prevalence of *H. pylori* [12]. Population genetics indicate that the Indonesian Malay *H. pylori* belongs to the hpEastAsia population and the hspMaori subpopulation of *H. pylori*. The Indonesian Malay *H. pylori* is highly similar to the *H. pylori* isolated from Manado [13] and Melanesians hspMaori [14] strains. These data suggest that the *H. pylori* originated from the Austronesian expansion in a recent infection, because the strain retains the genetic characteristics of its parental group. Since there was a similar ancestral history between Sundanese, Javanese, and Malays with a low *H. pylori* prevalence and no observation of specific Indonesian Malay *H. pylori*, we hypothesize that there is no separate *H. pylori* among the Indonesian Malay descendants. The currently reported *H. pylori* may have been imported following the intra-racial spread instead of inter-racial spread, as explained by the “Racial Cohort” hypothesis [11, 21].

We observed a high *H. pylori* prevalence among the Bataknese. The Bataknese descend from Austronesians who originated from Taiwan and the Philippines then migrated to North Sumatra via Java/Borneo. They settled mainly around the Great Toba Lake, which provided fresh water to support their agriculture activity [28]. Our observation confirmed a previous study showing a high prevalence of *H. pylori* in Bataknese [12, 29]. Interestingly, we observed low *H. pylori* prevalence among the Nias ethnic group, which is believed to have the same roots as the Bataknese. Even Bataknese from Medan tended to have a higher prevalence of *H, pylori* than people from Dolok Sanggul, Bataknese still commonly use boiled water as their primary drinking water source [12]. The water source is associated with *H. pylori* infection. This hypothesis was supported by the high *H. pylori* infection rate in the Japanese, who have drunk well water since before World War II [30, 31]. Our risk factor analysis among Indonesians from the five largest islands showed similar results [12]. This interesting distribution suggested that the main source of *H. pylori* among Bataknese might originate from Great Toba Lake.

We examined the *H. pylori* infection rate using five different diagnostic modalities. Even though we observed a different infection rate between the five tests, the inter-test essential agreement value and substantial Cohen’s Kappa value were good, suggesting valid results between tests and interchangeable usage. After dividing tests into invasive and non-invasive methods, we obtained better sensitivity and specificity for non-invasive methods compared with invasive tests, suggesting that the non-invasive methods were a better choice. Indeed, the ELISA is a favorable non-invasive method to detect *H. pylori* among clinicians. However, the ELISA has a bias toward current rather than past *H. pylori* infections; hence, infection should be confirmed with another diagnostic method. Other non-invasive methods, such as the stool antigen test (SAT) and ^14^C Urea Breath Test (UBT) showed promising performances; however, only a polyclonal antibody with low sensitivity for SAT is available in Indonesia [32]. ^14^C-UBT is widely available in Indonesia, but has not been locally validated yet.

Indonesia is widely known to have low *H. pylori* prevalence, but the number of dyspeptic patients is still high [12, 33]. The results of this study demonstrate that older age and infection with *H. pylori* contribute to worsening gastric mucosal conditions. Interestingly, *H. pylori* infection also associated with the development and recurrence of Henoch-Schönlein Purpura (HSP) after gastrointestinal manifestations [34]. However, we observed inflamed gastric mucosa in the absence of *H. pylori* in a previous study [35]. This finding suggests that causes other than *H. pylori* are responsible for the gastro-duodenal diseases. Future studies elucidating non-*H. pylori* microbes that responsible for gastro-duodenal diseases are necessary.

There were several limitations to our study. First, the sample number is relatively small, which may lead to weak statistical inferences. In addition, we measured *H. pylori* infection, which is usually established during childhood. Our study may have examined the factors leading to the continuation of the infected state, rather than the development of infection. We did not obtain the data that explains the low prevalence in the culture and habit among our participants. Therefore, a study to elucidate the cultural and habitual activities related to low *H. pylori* among Malay is needed.

## Conclusion

The prevalence of *H. pylori* infection among Malays is low, with no *H. pylori* in some ethnic groups, such as the Aceh. We also observed no *H. pylori* among the Javanese. Nevertheless, an exceptionally high *H. pylori* infection rate was observed among the Bataknese and a moderate infection rate was observed among the Nias. Even though the ethnic groups live together as a community, the *H. pylori* infection rate among Indonesian Malays descendant ethnic groups is extremely low, suggesting that no *H. pylori* originated among the Malays.

## Methods

### Sampling population and sample collection

We performed consecutive endoscopic surveys between January and February 2016 at 5 locations: 3 locations in Sumatra island, including Dolok Sanggul, Padang, and Palembang; 1 location in Nias Island, Gunungsitoli; and 1 location on Java Island, Cimacan. The mean age of the participants was 46.6 ± 14.5 and consisted of 96 males and 76 females. The ethnic groups were Bataknese (47), Javanese (2), Malay (36), Minang (32), Nias (33), and Sundanese (22). In addition, we analyzed 60 samples from our previous study [36]. These 60 samples originated from participants with a mean age of 41.6; 30 participants were male and 30 were female. The study inclusion criteria were patients who had a chronic dyspeptic symptom but already stopped proton pump inhibitor and antibiotic treatment outside of *H. pylori* eradication purposes for at least 2 weeks. The exclusion criteria were any history of *H. pylori* eradication therapy, partial/total gastrectomy, subjects with contraindication for endoscopic examination, and nonfasted patients. In addition, we collected patient demographics and history, including smoking and alcohol drinking habits by interview. Before taking a history and performing the upper endoscopic examination, we acquired written informed consent from all participants. Our current study protocol was approved by the ethics committees of Dr. Soetomo Teaching Hospital (Surabaya, Indonesia), Dr. Cipto Mangunkusumo Teaching Hospital (Jakarta, Indonesia), and Oita University Faculty of Medicine (Yufu, Japan).

We collected four gastric specimens for each patient during the endoscopic procedures. Three specimens were collected from the lesser curvature of the antrum, approximately 3 cm from the pyloric ring, which each of specimen was used for *H. pylori* culture, RUT, and histopathology examination, respectively. Another gastric specimen was taken from the greater curvature of the corpus, which was used only for histopathology examination. During the examination, the endoscopists also determined the gastric condition visually, including the presence of ulcers, inflammation of the gastric mucosa, and carcinoma. In addition, fasting serum was collected on the day of the endoscopy and stored at − 20 °C for ELISA.

### Evaluation for H. pylori infection

We evaluated the *H. pylori* infection status by a combination of five different methods, including culture, histology, IHC, RUT, and serology.

### Culture and rapid urease test

To culture *H. pylori*, one antral biopsy specimen was homogenized in saline and inoculated onto Mueller Hinton II Agar medium (Becton Dickinson, NJ, USA) supplemented with 7% horse blood without antibiotics. The plates were incubated for up to 10 days at 37 °C under microaerophilic conditions (10% O_2_, 5% CO_2_, and 85% N_2_). *H. pylori* were identified based on colony morphology, Gram staining results, and positive reactions for oxidase, catalase, and urease. Isolated strains were stored at -80 °C in Brucella Broth (Difco, NJ, USA) containing 10% dimethyl sulfoxide and 10% horse serum. For the RUT examination, the gastric specimen collected from the antrum was directly inserted into the RUT slide (CLO test, Kimberly-Clark, USA).

### Histology and immunohistochemistry examination

Biopsy specimens were stored in 10% buffered formalin then embedded in paraffin blocks. Serial sections were stained with hematoxylin and eosin and May-Giemsa stain. Stained samples were evaluated for *H. pylori* density using the updated Sydney system. The degree of bacterial density according to the updated Sydney system was: 0, normal; 1, mild; 2, moderate; and 3, marked [37]. Samples with bacterial density ≥ 1 were considered positive for *H. pylori*.

To increase the accuracy of detecting *H. pylori*, we also performed immunohistochemical staining. Briefly, tissue sections were incubated with anti-α-*H. pylori* antibody (DAKO, Glostrup, Denmark, product ID: B0471) overnight at 4 °C. After washing, the sections were incubated with biotinylated goat anti-rabbit IgG (Nichirei Co., Tokyo, Japan, product ID: 426011), followed by incubation with an avidin-conjugated horseradish peroxidase solution (Vectastain Elite ABC Kit; Vector Laboratories Inc., Burlingame, CA, USA). Peroxidase activity was detected using an H_2_O_2_/diaminobenzidine substrate solution [38]. The same experienced pathologist who analyzes for Myanmar, Vietnam, Bhutan, and the Dominican Republic evaluated all the specimens in this study to reduce the examiner bias [39–43].

### Serology evaluation

We measured the *H. pylori* antibody titers with an ELISA kit (Eiken, Co. Ltd., Tokyo, Japan, product ID: 4,987,026,182,711). The manufacturer’s recommended cut-off point for determining *H. pylori* infection was ≥ 10 U/mL. This cut-off point has been validated in the Indonesian population yielding a sensitivity and specificity of 66.7% and 97.2%, respectively [44].

Patients were considered to be negative for *H. pylori* infection when all five test results were negative, whereas patients with at least one positive test were considered positive for *H. pylori* infection.

### Determination of population genetics

We performed the population genetic analysis using the multilocus sequence typing (MLST) approach. Seven housekeeping genes of *H. pylori* (*atp*A, *efp*, *mut*Y, *ppa*, *trp*C, *ure*I, and *yph*C) were analyzed resulting in 3406 concatenated sequences. STRUCTURE version 2.3.4 [45] with no-admixture model algorithm was used against 2544 available MLST data on the pubMLST (https://pubmlst.org/). We used the parameter K = 7, as this number of K has been identified as the generated population genetics of *H. pylori* [14, 46, 47]. For the determination of the *H. pylori* subpopulation, we picked the hpEastAsia population only and ran the subsequent analysis of the STRUCTURE no-admixture model with K = 3, as previously described [14]. All of these analyses were carried out using Markov-Chain Monte Carlo of 1000,000 iterations with 100,000 burn-in. The phylogenetic tree was constructed using MEGA 7 [48] with Neighbor-Joining Tree [49] and Kimura-2 parameter substitution model [50], as these parameters were commonly used for phylogenetic analyses of *H. pylori*.

### Statistical analysis

Discrete variables were tested using the chi-square test; continuous variables were tested using the Mann–Whitney U and t-tests. The Saphiro–Wilk test was carried out for testing data distribution. A multivariate logistic regression model was used to calculate the odds ratios (OR) of the clinical outcomes and *H. pylori* infection by age, sex, and other demographic factors. The OR and 95% confidence intervals (CI) were used to estimate the odds. We also evaluated the consistency of the results between diagnostic modalities using Cohen’s Kappa test implemented in the irr package. A *P*-value of < 0.05 was accepted as statistically significant. All of these calculations were carried out on the R software version 4.0.3.

## Data Availability

The datasets used and/or analyzed during the current study are available from the corresponding author on reasonable request.

## Abbreviations

CI: Confidence intervals
MCMC: Markov-Chain Monte Carlo
OR: Odds ratios
SAT: Stool antigen test
UBT: Urea Breath Test
